# Supplementary material for: Cloning and Functional Analysis of TaWRI1Ls, the Key Genes for Grain Fatty Acid Synthesis in Bread Wheat
Source: Int J Mol Sci. 2022 May 10;23(10):5293. doi: 10.3390/ijms23105293 (PMC9141799; doi:10.3390/ijms23105293)
Supplement: Supplementary file 1 [file ijms-23-05293-s001.zip › Supplementary Figures.pptx]

## Slide 1
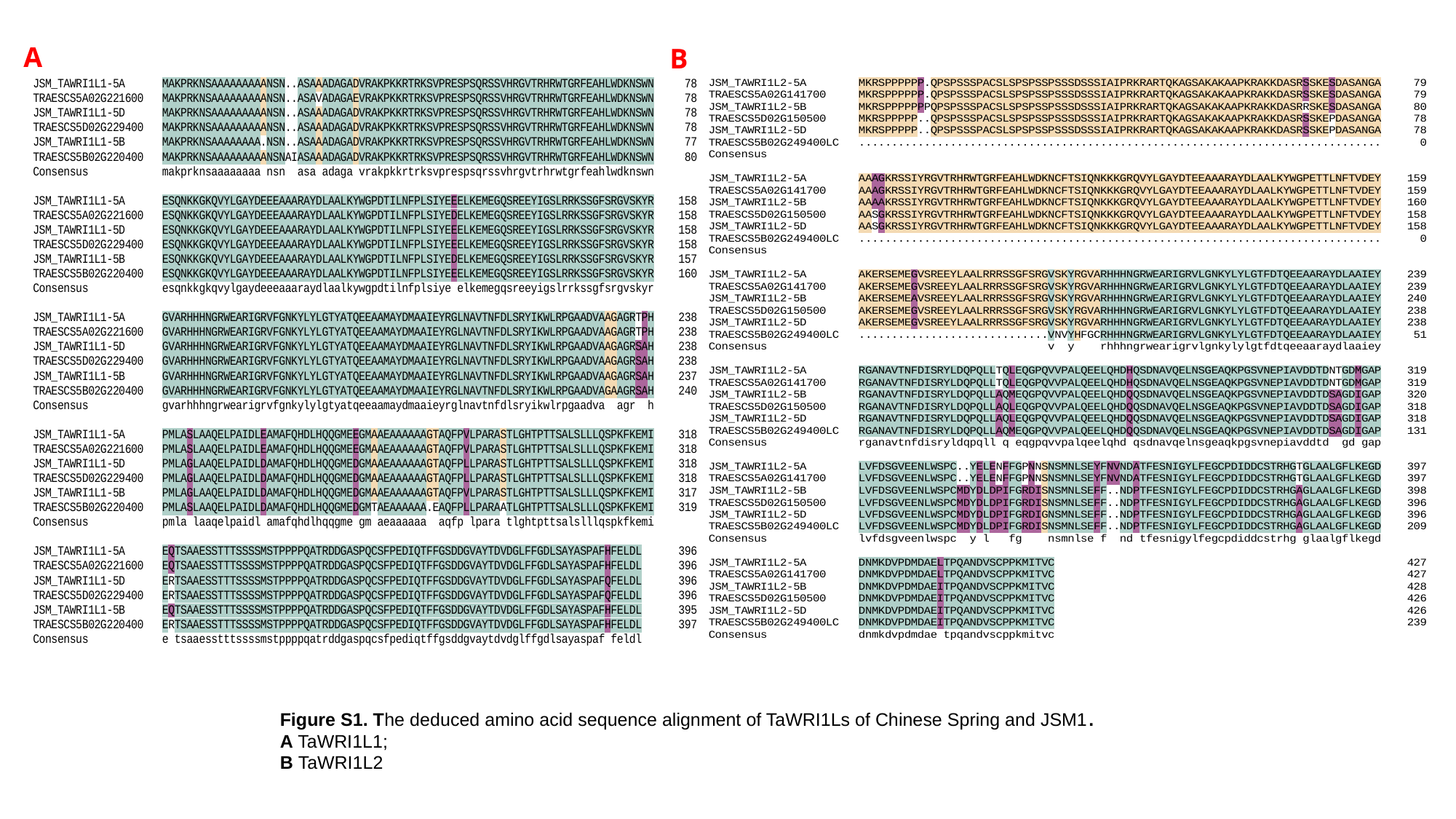

A
B
Figure S1. The deduced amino acid sequence alignment of TaWRI1Ls of Chinese Spring and JSM1.
A TaWRI1L1;
B TaWRI1L2

## Slide 2
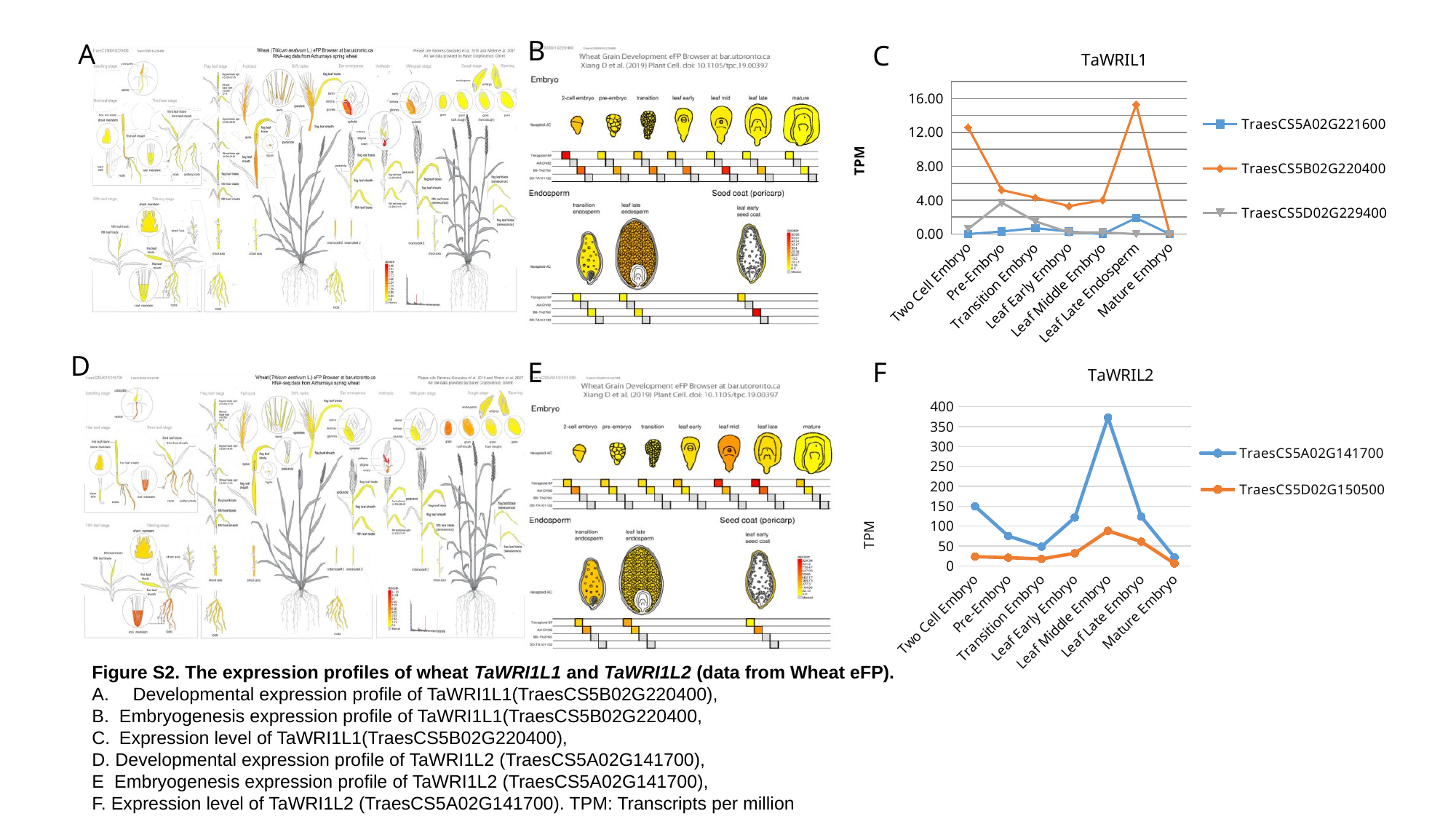

B
A
### Chart: TaWRIL1
| Category | TraesCS5A02G221600 | TraesCS5B02G220400 | TraesCS5D02G229400 |
|---|---|---|---|
| Two Cell Embryo | 0.0 | 12.6 | 0.6 |
| Pre-Embryo | 0.3 | 5.2 | 3.7 |
| Transition Embryo | 0.7 | 4.3 | 1.5 |
| Leaf Early Embryo | 0.3 | 3.3 | 0.2 |
| Leaf Middle Embryo | 0.0 | 4.0 | 0.2 |
| Leaf Late Endosperm | 1.9 | 15.3 | 0.0 |
| Mature Embryo | 0.0 | 0.0 | 0.0 |
### Chart: TaWRIL2
| Category | TraesCS5A02G141700 | TraesCS5D02G150500 |
|---|---|---|
| Two Cell Embryo | 150.1 | 23.2 |
| Pre-Embryo | 75.0 | 20.6 |
| Transition Embryo | 48.2 | 17.5 |
| Leaf Early Embryo | 121.5 | 31.7 |
| Leaf Middle Embryo | 372.9 | 88.1 |
| Leaf Late Embryo | 123.8 | 61.0 |
| Mature Embryo | 21.6 | 5.9 |
C
D
E
F
Figure S2. The expression profiles of wheat TaWRI1L1 and TaWRI1L2 (data from Wheat eFP).
Developmental expression profile of TaWRI1L1(TraesCS5B02G220400),
Embryogenesis expression profile of TaWRI1L1(TraesCS5B02G220400,
Expression level of TaWRI1L1(TraesCS5B02G220400),
D. Developmental expression profile of TaWRI1L2 (TraesCS5A02G141700),
E Embryogenesis expression profile of TaWRI1L2 (TraesCS5A02G141700),
F. Expression level of TaWRI1L2 (TraesCS5A02G141700). TPM: Transcripts per million
